# Knowledge and application of the ABCDEF bundle in a pediatric intensive care unit: a cross-sectional study

**DOI:** 10.1590/1984-0462/2026/44/2026069

**Published:** 2026-11-02

**Authors:** Aline Costa Fraga, Paula Maria Eidt Rovedder, Paulo Roberto Antonaccio Carvalho

**Affiliations:** a Universidade Federal do Rio Grande do Sul, Hospital de Clínicas de Porto Alegre, Graduate Program in Child and Adolescent Health, Porto Alegre, RS, Brazil.; b Universidade Federal do Rio Grande do Sul, Hospital de Clínicas de Porto Alegre, Graduate Program in Pulmonary Sciences, Porto Alegre, RS, Brazil.

**Keywords:** Delirium, Analgesia, Mechanical ventilation, Intensive care, Early mobilization, Patient care bundles, Delírio, Analgesia, Ventilação mecânica, Cuidados intensivos, Mobilização precoce, Pacotes de assistência ao paciente

## Abstract

**Objective:**

To describe healthcare professionals’ knowledge and use of the ABCDEF bundle in the Pediatric Intensive Care Unit (PICU) of Hospital de Clínicas de Porto Alegre (HCPA) using an online questionnaire.

**Methods:**

An analytical cross-sectional study was conducted among PICU healthcare professionals at HCPA. An online questionnaire assessed practices related to awakening and spontaneous breathing evaluation, medication selection and adjustment, delirium assessment, early mobilization, and family engagement. Perceived barriers and facilitators to the implementation of the ABCDEF bundle were also investigated.

**Results:**

A total of 71 questionnaires were analyzed. Participants were predominantly women (91.5%), with a mean age of 41 years; 23.9% were physicians; 42.3%, nursing technicians; and 33.8%, members of the multidisciplinary team. Only 25.4% reported knowing the ABCDEF bundle. The most frequently implemented components were medication adjustment (87.3%), family engagement (74.6%), pain and sedation assessment (60.6%), and early mobilization (60.5%). Delirium assessment is not performed according to 28.2% of respondents, while 21.1% were unsure whether it was conducted. Awakening testing was considered “not implemented” by 38% of participants, and spontaneous breathing trials were reported as “occasionally implemented” by 35.2%. No significant differences in bundle utilization scores were observed across professional categories, work shifts, or levels of education.

**Conclusions:**

The level of knowledge and implementation of the ABCDEF protocol is low and inconsistent among professionals working in the PICU. The implementation of delirium, sedation, and analgesia assessment and management, as well as spontaneous breathing trials, requires attention from health managers and researchers to standardize the provided care.

## RESUMO

**Objetivo:**

Descrever o conhecimento e utilização do *bundle* ABCDEF por profissionais de saúde da Unidade de Terapia Intensiva Pediátrica (UTIP) do Hospital de Clínicas de Porto Alegre (HCPA), utilizando um questionário online.

**Métodos:**

Foi realizado um estudo transversal analítico com profissionais de saúde da UTIP do HCPA. Um questionário online foi utilizado para verificar a avaliação do despertar e da respiração espontânea, a seleção e o ajuste de medicamentos, a avaliação do risco de delírio, a implementação da mobilização precoce e o envolvimento familiar. Também foram investigados as barreiras e os facilitadores percebidos para a implementação do *bundle* ABCDEF.

**Resultados:**

Foram analisados 71 questionários. Os participantes eram predominantemente do sexo feminino (91,5%), com idade média de 41 anos; 23,9% eram médicos, 42,3% eram técnicos de enfermagem e 33,8%, membros da equipe multidisciplinar. Apenas 25,4% relataram conhecer o *bundle* ABCDEF. Os componentes mais frequentemente implementados foram o ajuste dinâmico de medicamentos (87,3%), incentivo ao envolvimento da família nos cuidados (74,6%), avaliação da dor e sedação (60,6%) e mobilização precoce (60,5%). A avaliação do delírio não é realizada, segundo 28,2% dos respondentes, enquanto 21,1% não tinham certeza se era realizada. Os testes de despertar foram considerados “não implementados” por 38% dos respondentes, e os testes de respiração espontânea foram relatados como “implementados ocasionalmente” por 35,2%. Não foram observadas diferenças estatisticamente significativas nos escores de utilização do *bundle* entre diferentes categorias profissionais, turnos de trabalho ou níveis de escolaridade.

**Conclusões:**

O nível de conhecimento e aplicação do *bundle* ABCDEF é baixo e inconsistente entre os profissionais que atuam na UTIP. Além disso, a implementação da avaliação e manejo do delírio, do manejo da sedação e analgesia e dos testes de respiração espontânea requer atenção por parte de gestores e pesquisadores da área da saúde, visando à padronização dos cuidados prestados.

## INTRODUCTION

Critically-ill children admitted to the Pediatric Intensive Care Unit (PICU) are exposed to a stressful environment. Due to the nature of their underlying condition, sedatives and analgesics are often prescribed, especially for those requiring ventilatory support.^1^ However, the literature shows that inadequate management of sedation and analgesia can contribute to increased morbidity and mortality in pediatric patients,^2^ ^,^ ^3^ and may prolong the weaning process from mechanical ventilation.

In addition, physical mobility is often impaired in critically-ill patients, making exercise essential to prevent and rehabilitate intensive care unit-acquired weakness.^4^ Considering these impairments, the consolidation of care bundles - sets of interventions aimed at prioritizing and accelerating patients’ discharge from the ICU - should be a continuous effort by the multidisciplinary team.^1^ ^,^ ^4^ ^,^ ^5^

In the pursuit of earlier ICU discharge, Pandharipande et al.^4^ proposed a bundle for “assessment, prevention, and management of pain (A); daily awakening and spontaneous breathing trials (B); choice of analgesia and sedation (C); assessment, prevention, and management of delirium (D); early mobility and exercise (E),” along with item (F), family engagement and empowerment - known as the ABCDEF bundle.

Given this context, authors of recent studies suggest that the use of the ABCDEF bundle in the care of critically-ill pediatric patients may reduce mechanical ventilation time, PICU length of stay, and overall hospital stay as well as improve patients’ function.^5^ Although researchers indicate that full implementation of the ABCDEF bundle is associated with clinical benefits, it is often applied incompletely.^1^ ^,^ ^6^ ^,^ ^7^

Barnes-Daly et al.^8^ and Pun et al.^9^ observed that implementation of the ABCDEF bundle resulted in substantial improvements among adult ICU patients. Patients experienced more days alive and free from delirium and coma with both full and partial adherence to the protocol. Regarding its application in pediatric ICUs, Lin et al.^10^ found no reduction in mechanical ventilation duration or ICU length of stay. However, a significant association with reduced mortality was observed. In their study, each 10% increase in overall bundle adherence was associated with a 34% reduction in the odds of death. Furthermore, each daily 10% increase in adherence was associated with a modest reduction in the risk of daily mortality.

Considering the described benefits, analyzing the knowledge and practical use - even if informal - of the ABCDEF bundle by the healthcare team in the PICU could help assess the barriers and facilitators to its full implementation, identify areas for improving quality of care, reduce hospital costs, and optimize resource utilization. We hypothesized that healthcare professionals working in the PICU of the Hospital de Clínicas de Porto Alegre, in Brazil, are aware of the ABCDEF bundle components; however, these components are not consistently implemented as part of routine clinical care. Furthermore, we hypothesized that occupation, level of education, length of service in the PICU, and work shift could influence the bundle implementation score.

## METHOD

This analytical cross-sectional study was conducted in the PICU of the Hospital de Clínicas de Porto Alegre (HCPA), a tertiary teaching hospital in southern Brazil. The study followed the Strengthening the Reporting of Observational Studies in Epidemiology (STROBE) recommendations and the Checklist for Reporting Results of Internet E-Surveys (CHERRIES). The study was conducted from November 2022 to August 2023.

All healthcare professionals working in the PICU at HCPA during the study period were considered eligible for participation. The target population included physicians, nurses, nursing technicians, physiotherapists, nutritionists, psychologists, pharmacists, and social workers involved in patient care. Participants were recruited through convenience sampling.

The inclusion criteria were:

1. Being an active healthcare professional in the PICU; and
2. Providing patient care.

Professionals with less than three months of experience in the unit were excluded, as this period was considered insufficient for integration into the unit’s care routines. A total of 104 eligible professionals were invited to participate.^1^

This study was approved by the Institutional Research Ethics Committee (CAAE number: 58087822.2.0000.5327). All participants provided electronic informed consent prior to participation. The study complied with the ethical principles established by Resolution 466/2012 of the Brazilian National Health Council.

A closed web-based survey was developed using Google Forms. Access to the questionnaire was restricted to invited healthcare professionals through institutional email invitations and printed folders containing a QR code linking directly to the survey. The first page of the survey presented the study objectives, estimated completion time, voluntary nature of participation, confidentiality assurances, and the electronic Informed Consent Form. Participants could proceed to the questionnaire only after providing electronic informed consent.

The estimated time to fully complete the questionnaire was 15 minutes, and it consisted of 32 items distributed across 8 sections. The questionnaire was translated and adapted from the study by Huang et al.^1^ in order to better reflect the objectives of the present study. To prevent duplicate entries, the survey platform was configured to limit one response per participant through institutional account authentication. Participants were allowed to review and modify their responses before final submission. Responses were automatically recorded in a password-protected spreadsheet linked to the main investigator’s institutional Google Drive account. Because Google Forms does not provide information on the number of visitors who initiated the questionnaire without submitting it, view and participation rates could not be calculated.

Before dissemination, the adapted questionnaire underwent internal review by the research team to assess content clarity and applicability to the local context. No formal external pilot testing was conducted. The final questionnaire included mandatory fields for primary outcome variables to minimize missing data. Response duration was not used as an exclusion criterion.

The primary outcomes were: knowledge of the ABCDEF bundle (yes/no) and implementation score of each bundle component (4-point Likert scale) as never implemented=1, occasionally implemented [<25% of patients]=2, frequently implemented [25-75% of patients]=3, and generally implemented [>75% of patients]=4. If the professional was unsure whether the item was implemented, the option “I do not feel qualified to answer” was provided. Secondary variables included demographic and professional characteristics (age, sex, occupation, level of education, years of experience, and work shift).

For the implemented items, there was space to fill out the pain assessment scales, sedatives, analgesics used, delirium management, nonpharmacological interventions, early mobilization practices, and family involvement measures. At the end of the questionnaire, multiple-choice questions were included to identify the perceived barriers and facilitators to the full implementation of the ABCDEF bundle.

Data were analyzed using IBM SPSS Statistics for Windows, version 20.0 (IBM Corp., Armonk, NY, USA). Descriptive statistics were initially performed for all study variables. Categorical variables were presented as absolute and relative frequencies, whereas continuous variables were summarized using measures of central tendency and dispersion. The normality of continuous variables was assessed using the Shapiro-Wilk and Kolmogorov-Smirnov tests, along with visual inspection of histograms and normal Q-Q plots. Given the small sample sizes in several subgroups, unequal group distributions, and evidence of non-normality in some categories, nonparametric methods were adopted for inferential analyses, using the Mann-Whitney U test. Comparisons of ABCDEF bundle utilization scores, derived from responses on a 4-point Likert scale, across professional categories, work shifts, and levels of education were performed using the Kruskal-Wallis test.

Only participants with complete data for the variables included in each analysis were considered. All statistical tests were two-tailed, and statistical significance was set at p<0.05.

## RESULTS

Of 104 eligible professionals, 71 completed the questionnaire (response rate: 68.3%). No participants were excluded after submission. Nonresponders were not characterized. Missing data were minimal and limited to specific questionnaire items. We obtained 100% adherence among the multidisciplinary team (physiotherapists, nutritionists, psychologists, pharmacists, and social workers), followed by 80.9% of the medical team, 60% of nursing technicians, and 57.1% of nurses. The sample consisted predominantly of women (91.5%) (Table 1), and most participants (69%) had more than five years of PICU experience.

**Table 1.** Sociodemographic characteristics of participants, healthcare professionals working at a Brazilian Pediatric Intensive Care Unit (n=71).

Characteristic	n (%) or mean±SD*	Research adherence (%)
Sex		
Women	65 (91.5)	
Age (years)	41±8.7	
Occupation		
Medical team	17 (23.9)	80.9
Nurses	12 (16.9)	57.1
Nursing technicians	30 (42.3)	60
Physiotherapist	6 (8.5)	100
Nutritionist	2 (2.8)	100
Psychologist	2 (2.8)	100
Pharmacist	1 (1.4)	100
Social worker	1 (1.4)	100

Notes: *Values are presented as mean±standard deviation for continuous variables and n (%) for categorical variables. SD: standard deviation.

Source: Prepared by the authors.

The knowledge rate for the ABCDEF bundle was 25.4%, with physiotherapy and nutrition being the most familiar professional categories (100% of professionals), followed by the medical team (80.9%) (Table 1).

Implementation varied across components, with higher adherence to medication adjustment (87.3%) and family engagement in care (74.6%), and lower adherence to delirium assessment and awakening and spontaneous breathing trials (Table 2).

**Table 2.** Status of the implementation of awakening and breathing tests, medication choice, delirium management, and early exercise/mobility for healthcare professionals working at a Brazilian Pediatric Intensive Care Unit.

Component	Never (n/%)	Occasionally (n/%)	Frequently (n/%)	Generally (n/%)	Do not know (n/%)
Spontaneous awakening tests	27 (38.0)	17 (24.0)	8 (11.2)	2 (2.8)	17 (24.0)
Spontaneous breathing tests	16 (22.5)	25 (35.2)	13 (18.3)	2 (2.8)	15 (21.2)
Dynamic medication adjustment	1 (1.4)	3 (4.2)	16 (22.6)	46 (64.8)	5 (7.0)
Sedation assessment	0 (0.0)	17 (24.0)	11 (15.5)	32 (45.0)	11 (15.5)
Pain assessment	1 (1.4)	4 (5.7)	18 (25.3)	44 (61.9)	4 (5.7)
Delirium assessment	20 (28.1)	10 (14.0)	15 (21.2)	11 (15.5)	15 (21.2)
Early mobilization	3 (4.2)	18 (25.3)	17 (24.0)	26 (36.6)	7 (9.9)
Family involvement in care	3 (4.2)	13 (18.3)	28 (39.5)	25 (35.2)	2 (2.8)

Source: Prepared by the authors.

We found no statistically significant differences in bundle implementation scores across different professional categories, work shifts, and levels of education. However, the highest scores, meaning the professionals who most frequently reported implementing the ABCDEF bundle components, were verified in those with graduate degrees (Table 3).

**Table 3.** Bivariate analysis of the ABCDEF bundle behavior scores for healthcare professionals working at a Brazilian Pediatric Intensive Care Unit (PICU) (n=71).

Group	n (%)	Score (mean±SD)	p-value
Occupation			
Medical Team	17 (23.9)	16.64±3.62	0.260
Nurses	12 (16.9)	11.01±2.86	
Nursing Technicians	30 (42.3)	14.17±1.8	
Multidisciplinary Team*	12 (16.9)	16.5±1.7	
Level of education			
Higher Education	8 (11.2)	14.87±4.02	0.391
Technical Course	27 (38.0)	13.6±2.74	
Graduate degree^†^	36 (50.7)	16.61±3.52	
Length of service in the PICU			
Three months to two years	12 (16.9)	12.25±5.67	0.164
Two to five years	16 (22.5)	12.78±4.17	
Five to ten years	21 (29.5)	15±2.55	
More than ten years	22 (30.9)	15.17±2.75	
Work shift			
Morning	13 (18.3)	17.17±1.6	0.321
Afternoon	17 (23.9)	13.91±3.33	
Night	21 (29.6)	12.5±3.02	
On-call^‡^	7 (9.9)	12.2±1.64	
Full-time/Variable^§^	13 (18.3)	17.33±2.64	

Notes: *Physiotherapist, nutritionist, psychologist, pharmacist, social worker; ^†^Specialization, residency, master’s, and doctorate degrees; ^‡^Weekend/holiday coverage in full-time shift and some shifts during the week; ^§^Full-time shift during some weekdays or variable shifts according to the schedule. SD: standard deviation.

Source: Prepared by the authors.

Regarding delirium, 45.1% of participants reported no protocol for its assessment and management, and 84.5% did not know which tool was used to assess it. The most used medications for delirium management were haloperidol and quetiapine, and nonpharmacological interventions - such as noise and light reduction, cognitive stimulation, and clustering of procedures - were commonly reported.

Early mobilization, although marked as frequently or generally implemented in 60.5% of responses, does not follow a standardized assessment and intervention protocol. Ninety percent (90.1%) of professionals reported that mobility was not assessed or did not know how to respond, and only 2.8% mentioned the Functional Status Score for the Intensive Care Unit (FSS-ICU) as an assessment tool.

More than half of the professionals (74.6%) reported frequently promoting family engagement in patient care in this PICU. Of these, 53.5% also mentioned involving family members in delirium management.

The most cited barriers to implementing the ABCDEF bundle as part of routine care by professionals were related to patients, cultural factors, and work processes. Conversely, the fact that it is a teaching hospital and the availability of training opportunities were identified as facilitators (Table 4).

**Table 4.** Barriers and facilitators for the implementation of the ABCDEF bundle by healthcare professionals working at a Brazilian Pediatric Intensive Care Unit.

Barriers	n (%)
Patient instability	48 (67.6)
Limited knowledge, resistance to change, and communication difficulties within the team	29 (40.8)
Heavy workload and competing priorities	23 (33.8)
**Facilitators**	
Training opportunities and protocol development	66 (94.3)
Teaching hospital	57 (81.6)
Discussion during daily rounds	41 (57.7)

Source: Prepared by the authors.

## DISCUSSION

More than half of the participants (74.6%) were not even familiar with the ABCDEF bundle, a finding that may reflect the well-documented delay in the adoption of evidence-based interventions in pediatric care.^11^ This highlights the importance of continuing education programs and institutional training initiatives to strengthen professionals’ knowledge and facilitate the implementation of evidence-based practices in PICUs.

An analysis of the use of the ABCDEF bundle in adult patients showed a dose-response effect, with an increase in the effect dependent on the number of bundle items implemented.^12^ The implementation of the items promotes, with each 10% of integrated implementation, a 15% increase in the likelihood of survival and absence of delirium the following day.^8^ Although the bundle components are interrelated and exert synergistic effects, partial implementation is still preferable to no implementation.^12^ Authors of a recent international study reported full implementation of the bundle in only 9% of PICUs across the USA, Canada, Brazil, and Europe.^13^ In the present study, the most frequently implemented components were dynamic medication adjustment (87.3%), family engagement in care (74.6%), pain and sedation assessment (60.6%), early mobilization (60.5%), and delirium assessment (36.6%).

Regarding ventilator liberation, Bacci et al.^14^ found that 83.3% of Brazilian neonatal and pediatric ICUs used spontaneous breathing trials (SBTs) during weaning from mechanical ventilation, a finding consistent with a prospective cohort study from the United States reporting SBT use in 62% of PICUs.^15^ Despite their widespread use, weaning protocols considerably vary, reflecting the lack of standardized pediatric guidelines. In our study, 53.5% of professionals reported performing SBTs occasionally or frequently.

In contrast, spontaneous awakening trials remain less commonly adopted in pediatric ICU due to limited evidence supporting their effectiveness.^16^ A multicenter randomized controlled trial demonstrated that daily sedation interruption combined with sedation protocols did not improve clinical outcomes and was associated with higher mortality compared with sedation protocols alone.^17^ Nevertheless, further research is needed to clarify the role of spontaneous awakening trials within multidisciplinary pediatric critical care strategies.^16^ ^,^ ^17^ In our study, 38% of professionals reported not routinely performing this practice.

Colleti Junior et al.^16^ studied the use of clinical tools for sedation, analgesia, and delirium prevention assessment among pediatric intensivists in Brazil. The authors showed that pain and sedation are assessed in the clinical practice of pediatric intensivists, who reported following sedation and analgesia guidelines and protocols. This aligns with our study, where 92.8% and 84.4% of professionals reported routinely assessing pain and sedation, respectively.

Guerra et al.^18^ reported that 84% of pediatric intensivists in Canada use sedation/analgesia scores in their units, with the COMFORT (41%) and COMFORT-B (15%) scales being the most used. Kudchadkar et al.^19^ showed that, although 70% of questionnaire respondents reported having sedation scores in their PICUs, only 42% used them routinely to determine patient care goals.

Authors of several studies demonstrate that the development of delirium has been consistently associated with increased morbidity and mortality, prolonged mechanical ventilation, longer lengths of hospital stay, and higher healthcare costs.^20^ ^,^ ^21^ Although the importance of screening and management has been widely recognized, implementation remains limited worldwide. Huang et al.^1^ reported that 80.4% of PICU professionals in China never performed delirium screening, while a North American survey found routine assessment rates as low as 2%.^19^

In our study, the responses greatly varied: 28.2% of professionals reported never assessing delirium, and 50.7% reported assessing it, either occasionally, frequently, or generally. However, 84.5% of participants did not know which tool was used for its assessment, and 45.1% of participants reported that there was no protocol for delirium assessment and management.

Considering that children are subject to changes in language based on age, assessing their delirium is a challenge.^21^ In 2016, the European Society of Pediatric and Neonatal Intensive Care recommended the use of the Cornell Assessment of Pediatric Delirium (CAPD) tool to assess delirium in pediatric patients (level of evidence A),^22^ which has already been translated and adapted for the Brazilian population by Barbosa et al.^23^

Early mobilization was reported by 85.9% of professionals as being performed occasionally, frequently, or routinely. This finding is consistent with a multicenter Chinese study reporting an early mobilization prevalence of 80.3% in PICUs.^24^ However, mechanically ventilated patients were significantly less likely to receive mobilization interventions.^24^ Previous research has demonstrated that the implementation of mobilization protocols and staff training programs increases engagement among healthcare teams, patients, and families.^25^ Despite relatively high mobilization rates in our study, the absence of formal protocols suggests opportunities for greater standardization and optimization of rehabilitation practices. Family presence has also been identified as a key predictor of successful out-of-bed mobilization.^24^

In our study, 92.9% of professionals reported encouraging family engagement in patient care as well as in delirium management. Evidence indicates that family-centered care contributes to emotional support, improved comfort, better sleep quality, facilitation of mobilization activities, and reductions in delirium incidence and severity.^26^ ^,^ ^27^

There was no statistically significant difference in the bundle utilization score between different professional categories, work shifts, and levels of education. However, the highest scores, i.e., the professionals who most frequently reported implementing the ABCDEF bundle components, were those with graduate degree education (master’s, doctorate, specialization, and/or residency). As discussed by Huang et al.^1^ and Costa et al.,^6^ the intensive care environment requires continuous learning to update and acquire new knowledge and skills. Studies have shown that lack of knowledge and awareness is an important factor affecting the implementation of the ABCDEF bundle by professionals.

In our study, in addition to the lack of knowledge, patient instability, resistance to change, communication difficulties within the team, and heavy workload were the most cited barriers by professionals for implementing the ABCDEF bundle as part of routine care. Balas et al.^28^ and Costa et al.^6^ suggest that managers should be attentive to the professionals’ workload and the allocation of human resources to better implement the ABCDEF bundle. Furthermore, the extensive bureaucratic organization of care and lack of leadership may affect its implementation. According to these authors, good teamwork facilitates the implementation of the ABCDEF bundle and increases healthcare professionals’ trust.^6^ ^,^ ^28^ In our study, it was evident that being a teaching hospital, the opportunity to conduct training sessions and create protocols, as well as discussions during daily rounds, were mentioned as facilitators that could assist in the implementation of this tool.

This study has important strengths that support its internal validity. It included professionals from all multidisciplinary categories working in the PICU, with direct patient care responsibilities, providing a comprehensive assessment of ABCDEF bundle implementation. Satisfactory participation rates, particularly among physicians and allied health professionals, enhanced the representativeness of the findings within the study setting.

However, some limitations should be considered when interpreting the findings. First, the cross-sectional design does not permit causal inference about factors associated with ABCDEF bundle implementation. Moreover, the single-center design limits external validity. The use of self-reported data and a questionnaire not previously validated in Brazil may have introduced information and response bias. The prolonged data collection period and repeated recruitment efforts may have introduced selection bias. Moreover, small subgroup sizes reduced the statistical power and may have limited the detection of differences between groups.

Despite these limitations, the findings have important implications for pediatric intensive care practice. The low and inconsistent implementation of several ABCDEF bundle components underscores the need for institutional strategies to improve adherence and standardize care in PICUs. Educational initiatives, including multidisciplinary training, simulation-based learning, bedside teaching, and daily round discussions, may enhance healthcare professionals’ knowledge and facilitate implementation. Standardized protocols, validated pediatric assessment tools, leadership engagement, and multidisciplinary collaboration may further support implementation. Previous studies have shown that institutional support, clear team roles, staffing adjustments, and performance feedback are associated with greater bundle adherence.

Future research should evaluate multifaceted implementation strategies, including protocolized care pathways, educational interventions, and audit-and-feedback systems, to improve ABCDEF bundle adherence and their impact on clinical and patient-centered outcomes in pediatric critical care.

In conclusion, knowledge and implementation of the ABCDEF bundle remain limited and inconsistent among professionals working in the studied PICU. While pain assessment, sedation assessment, and family engagement are well-established, the implementation of delirium assessment, awakening and spontaneous breathing trials, and early mobilization remains variable. Neither occupation, level of education, length of service in the PICU, nor work shift were associated with the bundle implementation score. The main barriers for bundle implementation involved patient clinical status, interprofessional communication issues, and workload, whereas the main facilitators were training opportunities, local protocols, working at a teaching hospital, and discussions during daily rounds. These findings highlight the need for targeted implementation strategies to improve adherence and promote standardized pediatric intensive care practices.

## Data Availability

The database that originated the article is available with the corresponding author.
